# Pacemaker implantation in a patient with isolated persistent left superior vena cava using a delivery catheter: a case report

**DOI:** 10.1093/ehjcr/ytae031

**Published:** 2024-01-23

**Authors:** Toshinaru Kawakami, Kazuyuki Yahagi, Yu Horiuchi, Kengo Tanabe

**Affiliations:** Division of Cardiology, Mitsui Memorial Hospital, Kanda-Izumi-cho 1, Chiyoda-ku, Tokyo 101-8643, Japan; Division of Cardiology, Mitsui Memorial Hospital, Kanda-Izumi-cho 1, Chiyoda-ku, Tokyo 101-8643, Japan; Division of Cardiology, Mitsui Memorial Hospital, Kanda-Izumi-cho 1, Chiyoda-ku, Tokyo 101-8643, Japan; Division of Cardiology, Mitsui Memorial Hospital, Kanda-Izumi-cho 1, Chiyoda-ku, Tokyo 101-8643, Japan

**Keywords:** Persistent left superior vena cava, Right superior vena cava, Cardiovascular diseases, Permanent pacemaker implantation, Case report

## Abstract

**Background:**

Persistent left superior vena cava (PLSVC) with absent right superior vena cava, also termed ‘isolated PLSVC’, is extremely rare. Permanent pacemaker implantation in patients with isolated PLSVC is often difficult by the usual subclavian approach due to the unique anatomy. With the advent of delivery catheters in recent years, implantation using the same system has been reported.

**Case summary:**

A 47-year-old woman with symptomatic sick sinus syndrome was admitted to our institution for permanent pacemaker implantation. Preprocedural cardiac multidetector computed tomography (MDCT) showed isolated PLSVC. We performed pacemaker implantation successfully via the left subclavian approach, using the C315 delivery catheter system. The leads were stable on chest radiography, and the sensing and capture thresholds were unchanged. After the procedure, we integrated the delivery catheter images with cardiac MDCT using Ziostation, and they were well matched with the fluoroscopic images. At the 1-month follow-up, the patient was free of heart failure symptoms and had decreased levels of N-terminal prohormone of brain natriuretic peptide.

**Discussion:**

The C315 delivery catheter system was considered an option for permanent pacemaker implantation in patients with isolated PLSVC. When performing permanent pacemaker implantation in patients with unusual venous anatomy, integrating the delivery catheter images with cardiac MDCT allows for appropriate preoperative catheter selection.

**Learning points**

- To appreciate the role of diagnostic imaging, including venography, prior to permanent pacemaker implantation.
- To illustrate the usefulness of a delivery catheter when performing permanent pacemaker implantation in patients with unusual venous anatomy.

## Introduction

Persistent left superior vena cava (PLSVC) is the most common congenital anomaly of the thoracic venous system, occurring in 0.3–0.5% of the general population.^1^ Some patients with PLSVC (10–20%) lack the right superior vena cava (RSVC), as observed in this case, which is called isolated PLSVC.^2^ Persistent left superior vena cava is largely asymptomatic and incidentally detected during imaging, central venous catheterization, or cardiac device implantation. In isolated PLSVC cases, the proximity of the coronary sinus (CS) orifice to the tricuspid valve and the steep angle make it difficult to advance the right ventricular (RV) lead. With the recent advent of delivery catheters, implantation using the same system has been reported.^3^ We present a case of successful permanent pacemaker implantation in a patient with isolated PLSVC using the C315-S10 delivery catheter.

## Summary figure

**Table:** 

April 2014	The patient was diagnosed with sick sinus syndrome during an annual medical check-up. She was asymptomatic and followed up with a Holter electrocardiogram.
May 2022	The patient experienced worsening shortness of breath and decline from New York Heart Association functional class Ⅰ to Ⅱ. A Holter electrocardiogram showed a decreasing trend in the total heart rate to ∼55 000 beats per day.
August 2022	Chest radiography showed a gradual increase in the cardiothoracic ratio. Stress echocardiography showed an impaired heart rate response to exercise, and the patient was scheduled to receive permanent pacemaker implantation.
September 2022	Preprocedural cardiac multidetector computed tomography (MDCT) was performed to examine the coronary artery stenosis, and it incidentally showed isolated PLSVC.
25 January 2023	The patient underwent permanent pacemaker implantation. Lead placements were performed using catheter delivery systems. Laboratory data showed a slightly elevated level of N-terminal prohormone of brain natriuretic peptide (132 pg/mL).
2 February 2023	The patient was discharged without complications.
March 2023	At the 1-month follow-up, the patient was free of heart failure symptoms and had decreased levels of N-terminal prohormone of brain natriuretic peptide. Moreover, the leads were stable on chest radiography, and the sensing and capture thresholds were unchanged.

## Case presentation

A 47-year-old woman with a history of sick sinus syndrome, diagnosed 9 years previously, presented to our hospital. She experienced worsening shortness of breath and a decline from New York Heart Association functional class Ⅰ to Ⅱ. A Holter electrocardiogram showed a decreasing trend in the total heart rate to ∼55 000 beats per day. Chest radiography showed a gradual increase in the cardiothoracic ratio. Stress echocardiography showed an impaired heart rate response to exercise. Based on these findings, the patient was scheduled for permanent pacemaker implantation. On admission, her blood pressure was 109/66 mmHg, heart rate 37 b.p.m., and oxygen saturation 98% on room air. Physical examination revealed clear lung sounds, absent heart murmurs, and no lower extremity oedema.

Her medical history included diabetes mellitus and treated submucous uterine myoma.

The differential diagnosis for shortness of breath in a patient with sick sinus syndrome includes ischaemic heart and lung diseases.

**Figure 1:**
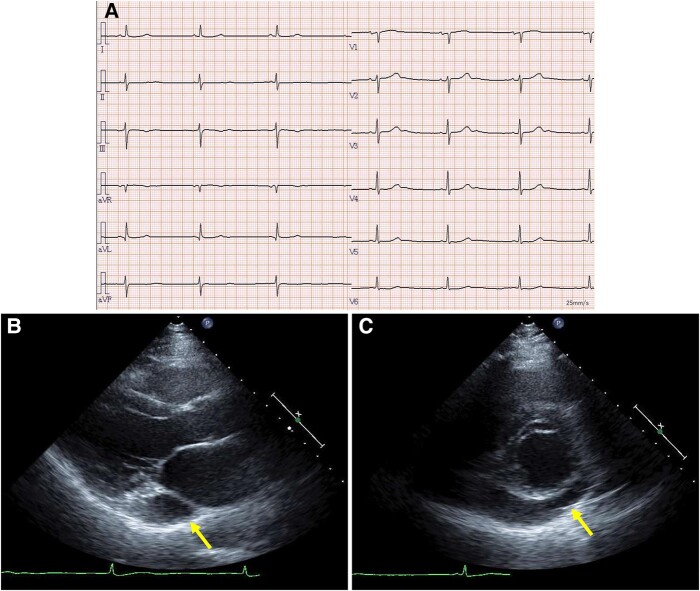
Electrocardiogram and transthoracic echocardiography. (*A*) The electrocardiogram revealed sinus bradycardia. Parasternal long-axis (*B*) and short-axis (*C*) views showing a dilated coronary sinus (arrows). Left ventricular ejection fraction was preserved with no asynergy.

**Figure 2:**
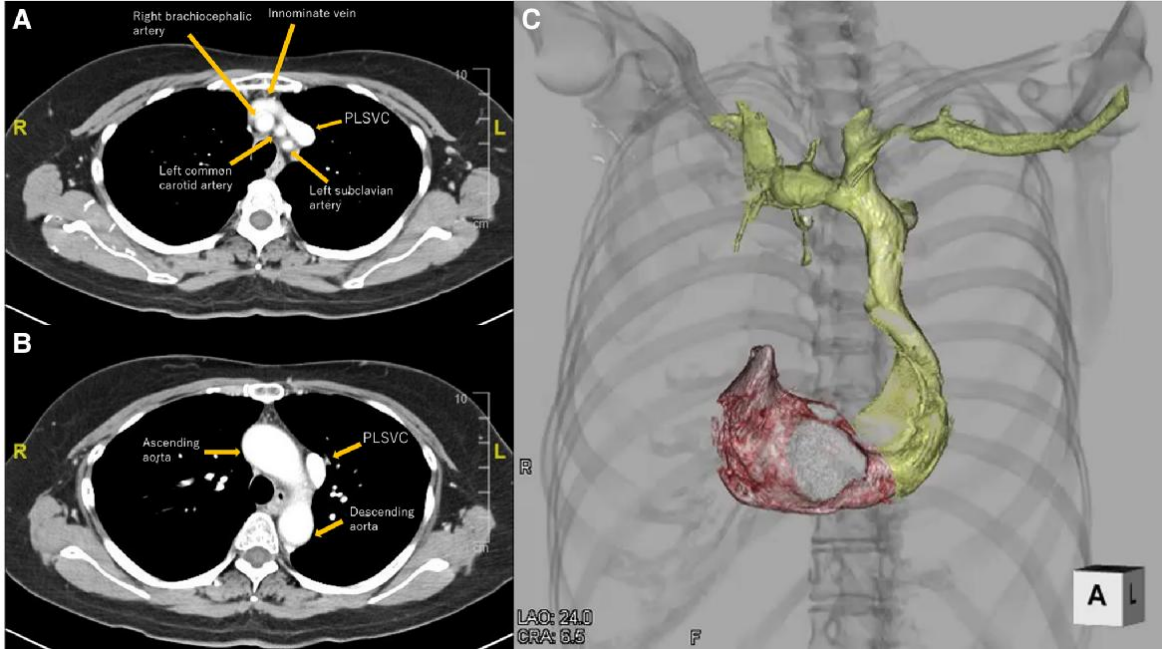
Cardiac computed tomography. (*A*) Axial view at the level of the superior margin of the sternum. (*B*) Axial view at the level of the sternal angle. (*C*) Reconstructed 3D image of the subclavian vein, persistent left superior vena cava, right atrium, and right ventricle. PLSVC, persistent left superior vena cava.

Laboratory data showed a slightly elevated level of N-terminal prohormone of brain natriuretic peptide (132 pg/mL, normal levels <55 pg/mL). Creatinine and electrolyte levels were normal. Chest radiography revealed no lung infiltration. The cardiothoracic ratio was 55%. Electrocardiography showed sinus bradycardia, at 43 b.p.m. (*Figure 1A*). Echocardiography revealed a dilated CS. The left ventricular ejection fraction was 71%, without significant valvular disease (*Figure 1B* and *C*). Preprocedural cardiac MDCT showed PLSVC with absent RSVC (*Figure 2*). Cardiac MDCT showed no significant coronary artery stenosis.

**Figure 3:**
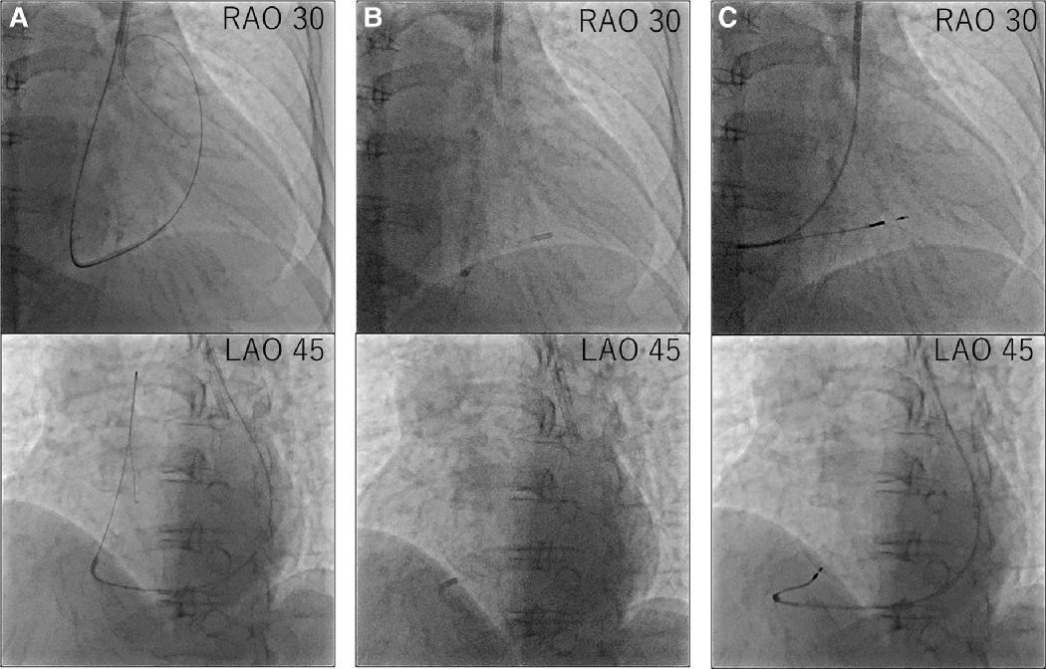
Procedural steps of right ventricular lead placement. (*A*) First, we delivered the guidewire to the pulmonary artery. (*B*) Second, we advanced the C315-S10 delivery catheter along the guidewire and then removed the guidewire. (*C*) Third, we delivered the right ventricular lead along the C315-S10 delivery catheter and screwed in the right ventricular lead.

**Figure 4:**
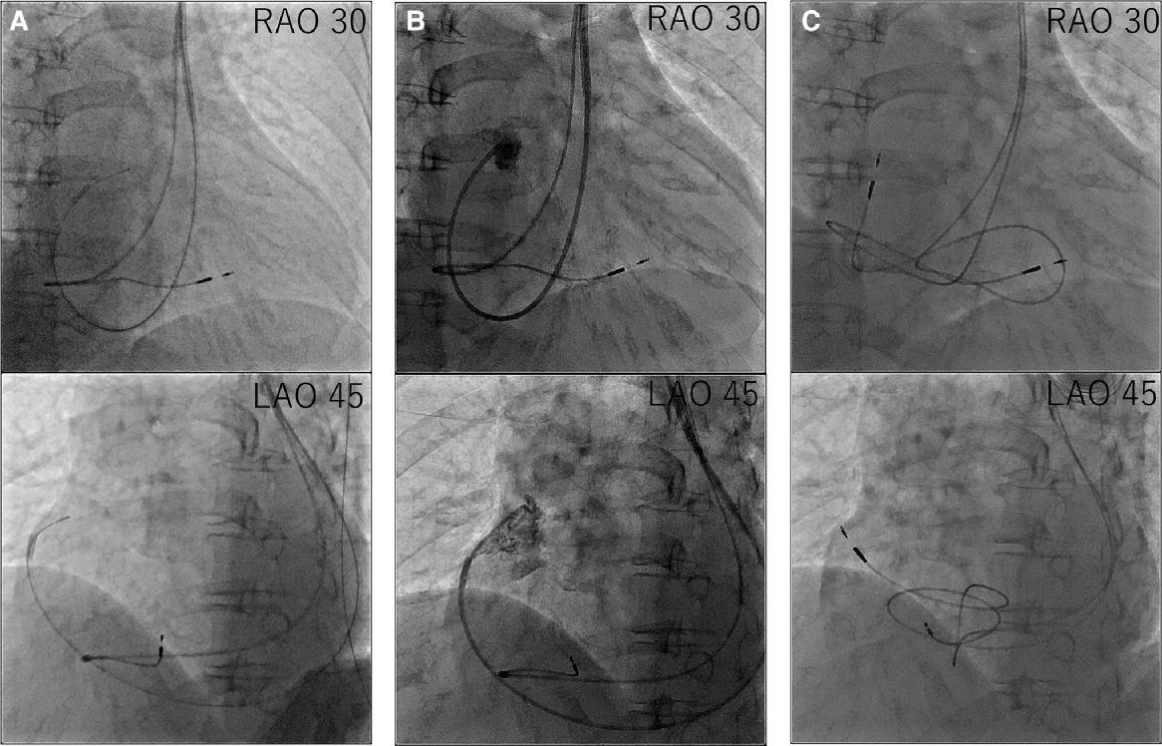
Procedural steps of right atrium lead placement. (*A*) First, we advanced the C315-H40 delivery catheter along the guidewire. (*B*) Second, we delivered a 4 Fr pigtail along the C315-H40 delivery catheter and performed right atrial appendage angiography. (*C*) Third, we delivered the right atrium lead along the C315-H40 delivery catheter and screwed in the right atrium lead.

Considering the correlation between the symptoms and electrocardiographic findings, the patient was scheduled for conventional permanent pacemaker implantation. The procedure was performed under local anaesthesia. Venography confirmed an absent RSVC and drainage of both subclavian veins into the right atrium (RA) via the enlarged CS. Owing to the absent RSVC, we opted for the left subclavian venous approach. First, we implanted an RV pacing lead through the left subclavian vein. When placing the RV lead via the CS, the steep angle between the CS ostium and tricuspid valve made it difficult to place the lead into the RV septum. We used a catheter delivery sheath (Model C315-S10, Medtronic, MN, USA), designed for the outflow tract or RV septum, and successfully introduced the sheath into the RV after performing right atrial angiography using a 4 Fr pigtail catheter, then screwed in SelectSecure™ 3830 lead (Medtronic) to the low septum of the RV (*Figure 3*). The sensing and capture thresholds were acceptable (sensed R-wave, 3.75 mV; threshold, 3.5 V/0.4 ms/bipolar). We then focused on RA pacing lead implantation. Initially, we used a stylet to manipulate the RA lead. However, we could not deliver the RA lead into the right atrial appendage. Therefore, we used a catheter delivery sheath (Model C315-H40, Medtronic) designed for the RV apex or triangle of Koch. The sheath was successfully advanced close to the right atrial appendage using a 4 Fr pigtail catheter. After radiopaque contrast was injected from the 4 Fr pigtail catheter to evaluate the right atrial appendage, the SelectSecure™ 3830 lead was screwed into the right atrial appendage (*Figure 4*). The lead showed acceptable sensing and capture thresholds (sensed P-wave, 0.75 mV; threshold, 5.0 V/1.0 ms). Both leads were connected to a pacemaker generator (Azure™ XT, Medtronic). The total time of the procedure was 259 min, and the radiation fluoroscopy lasted 60 min. A pacemaker check was performed 1 week after implantation. The leads were stable on chest radiography, and the sensing and capture thresholds were unchanged. The patient was discharged on Day 9 without complications.

At the 1-month follow-up, the patient was free of heart failure symptoms and had decreased levels of N-terminal prohormone of brain natriuretic peptide. Moreover, the leads were stable on chest radiography, and the sensing and capture thresholds were unchanged.

## Discussion

Persistent left superior vena cava is a vein that opens into the RA via the CS after the confluence of the left internal jugular vein and left subclavian vein following the failure of normal development of the left common vena cava. Persistent left superior vena cava is a venous anomaly that affects 0.3–0.5% of the general population and is associated with 1.3–4.5% of all congenital heart diseases.^1^ Persistent left superior vena cava is the most frequently observed thoracic venous malformation. Most affected patients are clinically asymptomatic and often diagnosed incidentally during the insertion of a cardiac pacemaker or central venous catheter through the left subclavian vein. In 92% of PLSVC cases, venous blood flows into the RA via the CS and 8% flows into the left atrium,^2^ which can cause a right–left shunt. Most cases are asymptomatic and never cause haemodynamic problems; however, in cases with right–left shunting, cyanosis, air embolization during catheterization, arrhythmias, cardiac arrest, coronary emboli (including angina pectoris), shock, and other serious complications may be reported. Isolated PLSVC has been observed in approximately one-tenth of all patients with PLSVC.^4^ In isolated PLSVC, permanent pacemaker implantation using the RV lead should be performed through the left subclavian vein and left superior vena cava via the CS; however, the steep angle between the CS ostium and tricuspid valve often makes lead placement difficult. It has been reported that lead placement is possible using a C- or U-shaped stylet with a stronger curve than usual.^1^ For decades, only the stylet system was available for lead placement. Recently, a new lead placement method, the catheter delivery system, was introduced. These catheters have specific 3D curve and facilitate lead placement in the RV septum.^5^ In the present case, we could easily place the RV lead using the C315-S10 catheter delivery system. The C315-H40 delivery catheter could also be an option for RA lead implantation. When the CS is enlarged on echocardiography, PLSVC should be strongly considered, and other imaging modalities, such as venography and MDCT, should be performed before pacemaker implantation.

**Figure 5:**
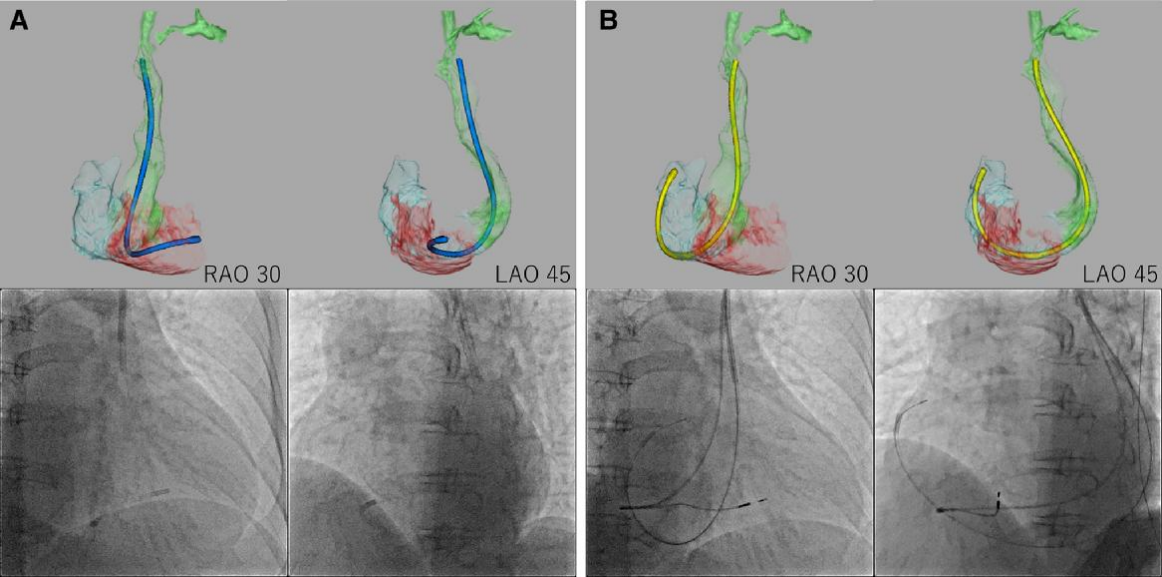
Delivery catheter merged with cardiac multidetector computed tomography. We imported the delivery catheter data and reconstructed them into 3D computed tomography, then merged them with the patient’s cardiac 3D multidetector computed tomography. (*A*) The distal end of the C315-S10 delivery catheter was in a similar position relative to the right ventricular septum between the 3D CT image and the fluoroscopic image. (*B*) The distal end of the C315-H40 delivery catheter was in a similar position relative to the right atrial appendage between the 3D computed tomography image and fluoroscopic image.

We propose a method for selecting an optimal delivery catheter for delivering pacemaker leads to the RA and RV by analysing cardiac MDCT using Ziostation (Ziosoft, Tokyo, Japan). We curved the delivery catheter and adjusted its shape using the 3D MDCT image as a reference. Then, we imported the delivery catheter data and merged it with the 3D MDCT (*Figure 5*).

In conclusion, we have presented a case of permanent pacemaker implantation in a patient with isolated PLSVC. The C315 delivery catheter system was considered an option for permanent pacemaker implantation in patients with isolated PLSVC. When performing permanent pacemaker implantation in patients with unusual venous anatomy, integrating the delivery catheter images with cardiac MDCT allows for more specific, efficient, and safe catheter selection.

## Data Availability

The data underlying this article will be shared upon reasonable request to the corresponding author.

## Lead author biography

Toshinaru Kawakami, MD, is a cardiologist at Mitsui Memorial Hospital, Tokyo, Japan. He graduated from the University of Tokyo Faculty of Medicine in 2018 and currently works as a chief fellow at Mitsui Memorial Hospital, Tokyo, Japan, since April 2022.

## References

[1] Li T, Xu Q, Liao HT, Asvestas D, Letsas KP, Li Y. Transvenous dual-chamber pacemaker implantation in patients with persistent left superior vena cava. BMC Cardiovasc Disord 2019;19:100. 31035937 10.1186/s12872-019-1082-7 PMC6489345

[2] Laasri K, El Graini S, Zahi H, Halfi IM, Bachri H, Massri EA, et al Persistent left superior vena cava: case report. Radiol Case Rep 2022;18:79–85. 36324842 10.1016/j.radcr.2022.09.076 PMC9619296

[3] Hiruma T, Nagase T, Mabuchi K, Ishiguro M, Seki R, Asano S, et al Pacemaker implantation using the SelectSecure system for a patient with persistent left superior vena cava and absent right superior vena cava: insights into techniques for stable lead fixation. J Arrhythm 2021;37:1105–1107. 34386139 10.1002/joa3.12581 PMC8339083

[4] Povoski SP, Khabiri H. Persistent left superior vena cava: review of the literature, clinical implications, and relevance of alterations in thoracic central venous anatomy as pertaining to the general principles of central venous access device placement and venography in cancer patients. World J Surg Oncol 2011;9:173. 22204758 10.1186/1477-7819-9-173 PMC3266648

[5] Worsnick SA, Sharma PS, Vijayaraman P. Right ventricular septal pacing: a paradigm shift. J Innov Card Rhythm Manag 2018;9:3137–3146. 32494491 10.19102/icrm.2018.090501 PMC7252807

